# RETRACTION: Effect of Powdered Vancomycin on Stopping Surgical Site Wound Infections in Neurosurgery: A Meta‐Analysis

**DOI:** 10.1111/iwj.70466

**Published:** 2025-04-02

## Abstract

**RETRACTION:** TianB. , HeY. , HanZ. , LiuT. , and ZhangX. , “Effect of Powdered Vancomycin on Stopping Surgical Site Wound Infections in Neurosurgery: A Meta‐Analysis,” International Wound Journal 20, no. 4 (2023): 1139–1150, 10.1111/iwj.13973.36237125 PMC10031230

The above article, published online on 13 October 2022, in Wiley Online Library (http://onlinelibrary.wiley.com/), has been retracted by agreement between the journal Editor in Chief, Professor Keith Harding; and John Wiley & Sons Ltd. Following an investigation by the publisher, all parties have concluded that this article was accepted solely on the basis of a compromised peer review process. The editors have therefore decided to retract the article. The authors did not respond to our notice regarding the retraction.

**RETRACTION:** B. Tian , Y. He , Z. Han , T. Liu , and X. Zhang , “Effect of Powdered Vancomycin on Stopping Surgical Site Wound Infections in Neurosurgery: A Meta‐Analysis,” International Wound Journal 20, no. 4 (2023): 1139–1150, 10.1111/iwj.13973.36237125 PMC10031230

The above article, published online on 13 October 2022, in Wiley Online Library (http://onlinelibrary.wiley.com/), has been retracted by agreement between the journal Editor in Chief, Professor Keith Harding; and John Wiley & Sons Ltd. Following an investigation by the publisher, all parties have concluded that this article was accepted solely on the basis of a compromised peer review process. The editors have therefore decided to retract the article. The authors did not respond to our notice regarding the retraction.

